# Introducing the OUTPACE Framework for Health Care Quality Improvement

**DOI:** 10.1161/CIRCOUTCOMES.125.012211

**Published:** 2025-10-21

**Authors:** Michele Bolles, Heather M. Alger, Mitchell S.V. Elkind, Howard Haft, Sabra C. Lewsey, Mariell Jessup, Karen E. Joynt Maddox, Chiadi E. Ndumele, Clyde W. Yancy, Christine Rutan, Michelle Congdon, Katherine Overton, Lynn Serdynski, Kathie Thomas, Gregg C. Fonarow

**Affiliations:** 1 American Heart Association, Dallas, TX (M.B., M.S.V.E., M.J., C.R., M.C., K.O., L.S., K.T.).; 2 Anumana, Inc, Cambridge, MA (H.M.A.).; 3 Department of Neurology, Vagelos College of Physicians and Surgeons, and Department of Epidemiology, Mailman School of Public Health, Columbia University, New York, NY (M.S.V.E.).; 4 University of Maryland School of Medicine, Baltimore (H.H.).; 5 Division of Cardiology Advanced Heart Failure/Transplant, Johns Hopkins University School of Medicine, Baltimore, MD (S.C.L., C.E.N.).; 6 Washington University School of Medicine and School of Public Health, Washington University Center for Advancing Health Services, Policy and Economics Research, St. Louis, MO (K.E.J.M.).; 7 Division of Cardiology, Feinberg School of Medicine, Northwestern University, Chicago, IL (C.W.Y.).; 8 Division of Cardiology, University of California, Los Angeles (G.C.F.).

**Keywords:** atrial fibrillation, cardiovascular diseases, coronary artery disease, heart failure, implementation science, quality improvement, stroke

## Abstract

Equitable, timely, and evidence-based care remains a central goal across health care ecosystems, yet significant quality gaps, care variability, and health disparities persist. Professional societies, including the American Heart Association, have long developed clinical practice guidelines to provide standardized, evidence-based recommendations across the cardiovascular care continuum. These guidelines are operationalized into quality measures to monitor care, identify gaps, and guide improvement. Professional societies, agencies, and health systems have applied implementation science strategies, such as education, data sharing, and evaluation, to improve care quality and achieve quality measures defined in the clinical practice guidelines. American Heart Association’s Get With The Guidelines programs target inpatient quality measures for stroke, heart failure, atrial fibrillation, resuscitation, and coronary artery disease, complemented by ambulatory quality improvement programs to support seamless care transitions. Decades of Get With The Guidelines implementation have enabled American Heart Association teams and volunteers to refine these programs, improving guideline adherence at local, regional, and national levels. Lessons learned informed the development of the Observe, Uncover, Trial, Personalize, Accelerate, Check, Expand Framework, designed to guide successful quality improvement initiatives. While existing quality improvement frameworks provide structured approaches, many are costly, slow, or siloed, limiting rapid-cycle, data-driven innovation across diverse health systems. The Observe, Uncover, Trial, Personalize, Accelerate, Check, Expand framework addresses these limitations as an adaptable model, applicable across care settings, disease areas, patient populations, system size, budgets, and target end points. Here, we illustrate the Observe, Uncover, Trial, Personalize, Accelerate, Check, Expand framework through 2 recent American Heart Association programs: Target: Aortic Stenosis and the IMPLEMENT-HF initiative, demonstrating its utility in guiding effective, scalable quality improvement.

Since 1980, the American Heart Association (AHA), the American College of Cardiology, and other professional societies have collaboratively developed clinical practice guidelines that provide standardized, graded, and evidence-based recommendations for clinicians treating patients across the care continuum from prevention to medical management of advanced cardiovascular conditions.

AHA is a longstanding leader in the development and execution of high-performing cardiovascular quality improvement (QI) initiatives. The Get With The Guidelines (GWTG) Program launched in 2000 as a pilot program focused on clinician education, technology solutions, and performance benchmarking to achieve adherence to clinical practice guidelines. GWTG has grown from the original pilot program with 24 hospitals and 1738 patient encounters to >2800 US hospitals and >1300 outpatient clinics in 2024. GWTG has served as the standard bearer in ensuring optimal guideline-based inpatient clinical care for >14 million patients hospitalized for stroke, heart failure, atrial fibrillation, resuscitation, and coronary artery disease across the United States. In addition to the 5 flagship inpatient GWTG programs, AHA has ambulatory QI programs and a comprehensive outpatient registry focused on optimizing cardiovascular care for patients. In 2023, these ambulatory programs were used by health care organizations to improve care delivery and clinical outcomes for >37 million patients (unpublished AHA metrics).

Building on decades of success and millions of lives positively impacted by AHA’s QI programs, volunteer clinicians and experts in implementation science joined together with AHA leadership to develop the Observe, Uncover, Trial, Personalize, Accelerate, Check, Expand (OUTPACE) framework to guide the development, structure, implementation, and evaluation of QI programs across a range of disease conditions (Figure).

**Figure.**
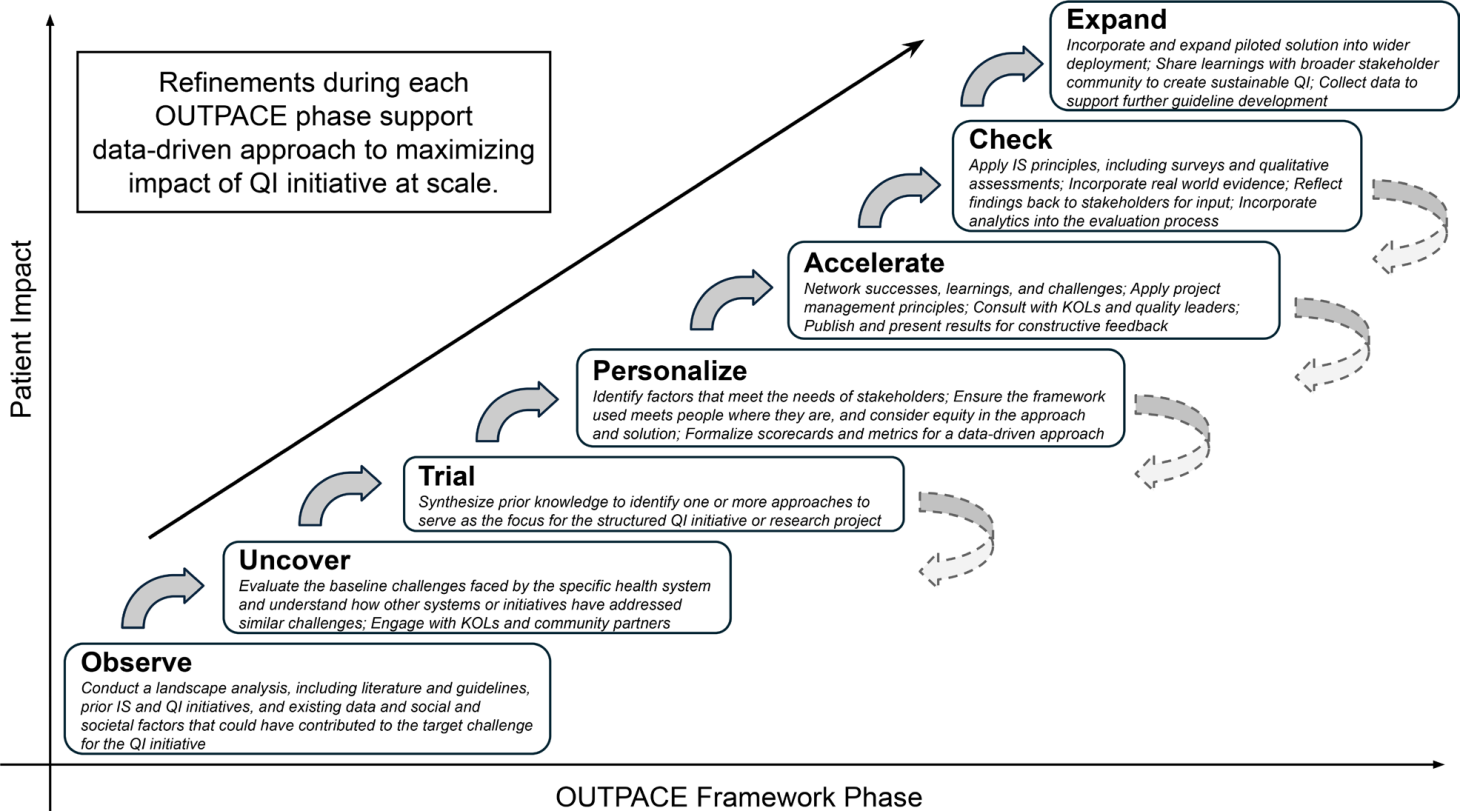
**Observe, Uncover, Trial, Personalize, Accelerate, Check, Expand (OUTPACE) framework for health care quality improvement (QI).** Solid arrows indicate advancement to the next phase; dashed arrows indicate optional return to prior phase(s). IS indicates implementation science; and KOL, key opinion leader.

## Local Challenges in Implementation

AHA recently led 2 nationwide initiatives coinciding with the development of OUTPACE: Target: Aortic Stenosis^1^ (TAS; Figure S1) and IMPLEMENT-HF^2^ (I-HF; Figure S2). We use the experiences with TAS and I-HF to illustrate the use of OUTPACE in a real-world example.

TAS aims to close care gaps along the journey of a patient with aortic stenosis by shortening time to definitive therapy, using a science advisory group and learning collaborative to identify effective workflows, analyze data, and share scalable improvement models. I-HF seeks to increase healthy days at home for patients with heart failure by optimizing guideline-directed medical therapy, assessing social needs, and addressing adherence barriers. Like TAS, I-HF employs a learning collaborative, data-driven approach with advisory group oversight to identify care gaps, improve workflows, and share models for performance improvement.

Both initiatives are examples of programs which applied principles that became components of the OUTPACE framework, as detailed in Figures S1 and S2, by observing the current state of care including data adherence and workflows, uncovering gaps leading to missed care opportunities, trialing new measures and process change strategies, personalizing each clinical setting’s improvement opportunities, and sharing solutions and models for accelerated measure improvement. Both initiatives monitored (checked) improvement through data analysis and learning collaborative discussions at a regular cadence, which helped inform a national education framework to expand initiative insights to a national audience.

The OUTPACE framework supports returning and repeating prior stages building on the experience and learnings at each stage. Specifically, OUTPACE follows a strategy of rapid-cycle innovation to pilot and pivot QI program efforts to optimize and iterate success before committing to a larger scale initiative. While this model was developed through the lens of CVD QI, its nimble and replicable framework allows OUTPACE to be adapted across a broad and diverse range of care settings.

## Design of the Initiative

QI programs such as GWTG or the programs developed by the Agency for Healthcare Research and Quality provide quality measures (QMs) to benchmark health care outcomes across the country. Data collected for these QMs are used to monitor national trends in health care, real-world adoption of clinical practice guidelines, and opportunities to address disparities in health care and outcomes.^3^

The Model for Improvement^4^ is a well-established framework that begins with 3 foundational questions to define the end point: What are we trying to accomplish? How will we know that a change has made an improvement? and What changes can we make that will result in improvement? These questions guide the plan-do-study-act cycle, a structured, evidence-based process for identifying, pilot-testing, and evaluating QI measures in health care.^4^ While effective, these standard approaches present an opportunity to adapt implementation science strategies for use at smaller scales or in resource-constrained settings, such as those limited by time, funding, experience, or capacity, where traditional models may be more difficult to apply.

OUTPACE is not intended to replace familiar processes, such as plan-do-study-act or learning health system models. Rather, it represents a natural evolution of these approaches, designed to address real-world limitations of applying these foundational models in complex health care settings.

Importantly, OUTPACE emphasizes rapid-cycle innovation and a focus on responding to local challenges. The framework incorporates expert consultation, piloting, learning collaboratives, interim evaluations, and continuous process improvement while focusing on innovative solutions to local challenges.

For example, systems experiencing large gap-to-goal challenges may benefit from a learning collaborative model, which brings together diverse expertise of stakeholder subject matter experts to network with systems that have previously overcome similar challenges or are piloting novel methods to tackle these challenges within their system.^5^ In this way, OUTPACE helps health systems prioritize their limited resources, iteratively test strategies, and evaluate interim outcomes before committing to full-scale deployment, thereby ensuring that solutions are both evidence-based and responsive to local needs.

## Implementation of the Initiative

The principles of OUTPACE are summarized in the Figure and detailed in the following. Although it loosely aligns with the plan-do-study-act cycle, OUTPACE is not strictly sequential; stages may be revisited to integrate new expertise and insights. This iterative design enables adaptation based on multimodal information gathering and observation across diverse health care settings. OUTPACE represents an evolution of QI approaches, synthesizing lessons learned from prior initiatives. The efforts of TAS and I-HF are used to illustrate their application in real-world settings and to demonstrate how OUTPACE can be used to build, refine, and optimize QI programs (Figures S1 and S2).

### Observe

In the observe phase, users identify target problems or end points for the implementation science program, conduct landscape analysis including review of current literature and guidelines, and assess prior QI initiatives that focused on similar end points. Importantly, OUTPACE users must consider gaps in knowledge that could contribute to health disparities.^3^ During this process, OUTPACE users should consider whether systematic societal or social factors may have contributed to the success or failure of prior initiatives and whether environmental factors should be considered in planning for the QI initiative (eg, emerging public health needs).

### Uncover

Uncover emphasizes the expertise of leaders, partners, and innovators as key stakeholders. Program developers engage thought-leaders to codevelop site selection rubrics that evaluate candidate sites’ readiness to implement QI programs, the diversity of target patient populations, and the feasibility of the target approaches.

### Trial

Trial synthesizes knowledge from observe with the opportunities identified in the uncover phase to design an evidence-based QI initiative. It may take the form of a small-scale pilot to pressure-test the initiative and assess whether target signals are detectable and replicable, or it may involve scaling up prior pilot studies.

### Personalize

Personalize is essential to all QI initiatives, incorporating stakeholder feedback to ensure that the proposed initiative addresses the specific needs of health systems and barriers to patient access. This phase emphasizes equity, helping avoid unintentional consequences that could disproportionately impact certain populations. Tools such as scorecards can guide site selection to ensure that participating health systems reflect the diversity of patient populations and care settings. To maximize effectiveness and equity, the personalize phase may require repeated cycles of refinement.

### Accelerate

Accelerate provides opportunities to evaluate the QI initiative and collaboratively interpret the pilot results, limitations, conclusions, and implications. This phase leverages the experience of similar programs, QI teams, and project management expertise. If early signals indicate the need for further optimization, program leaders may revisit earlier OUTPACE stages to refine before scaling.

### Check

In check, initiatives gather insights through real-world evidence, participant surveys, and statistical analysis of collected data. This phase is critical to determining both the effectiveness of the pilot (quantitative methods) and the acceptability of the QI strategy to implementers (qualitative methods). Based on these findings, program leaders may proceed to the expand phase or choose to return to earlier OUTPACE stages for further optimization before scaling.

### Expand

Expand is the final phase of OUTPACE and should begin only once QI initiative leaders, learning collaborative members, and expert consultants determine that check results are sufficiently robust to justify scaling from pilot evaluation to broader implementation. This phase emphasizes evaluating data to ensure equitable adoption and impact across patient populations. Comprehensive training and education across stakeholder groups are essential for the success of expand, the OUTPACE framework, and QI initiatives more broadly.

## Success of the Initiative

The OUTPACE framework was developed as a unique and replicable QI process, based on expertise acquired through years of leading local and national initiatives in the realms of QI and implementation science. Through this work, AHA’s QI programs have identified several themes and challenges that can impact the outcomes and success of local QI programs to meet their stated goals, including implementation challenges, heterogeneous approaches, and patient population considerations.

AHA’s I-HF QI initiative, conducted within GWTG-HF, aimed to improve outcomes for patients with heart failure (Figure S2). Over 3 years, 111 clinical centers across 7 regions focused on their most challenging QMs and systemic barriers, demonstrating substantial quarter-over-quarter improvements in GDMT adherence at discharge and 30 days post-discharge.^²^ Observations from local I-HF pilot studies mirror the principles described in OUTPACE. For example, the steps in one system’s local implementation of the I-HF program can be mapped onto OUTPACE: establishing baseline GDMT adherence (observe), convening cross-functional teams to identify challenges and opportunities (uncover), piloting interventions such as inpatient GDMT quad therapy and an outpatient GDMT order set (trial), and providing targeted education and resources (personalize). Over a 3-month pilot, the system monitored adherence improvements (check), laying the foundation to scale the program toward a 100% system-wide adherence goal for the following year. Another system’s I-HF implementation improved SDOH assessment rates from <5% to ≈70% by reviewing electronic health record tools (uncover), training abstractors (trial), collaborating with informatics (uncover), enhancing communication (personalize), and expanding from departments (accelerate) before ultimately scaling system-wide (AHA data on file).

## Translation to Other Settings

Although developed with a focus on cardiovascular outcomes, OUTPACE can be applied across health care challenges in inpatient, ambulatory, emergency care, community health, and public health settings. TAS and I-HF (Figures S1 and S2) illustrate its flexibility to test and refine QI initiatives across the care continuum and in any domain with measurable QMs. Before TAS, no systematic efforts had assessed the quality of care of aortic stenosis from diagnosis to treatment.^1^ TAS engaged diverse clinical sites across 5 regions to pilot and iterate QMs through OUTPACE-aligned rapid-cycle innovation before scaling nationwide (Figure S2).

For health systems struggling to meet QI goals, facing resource constraints, or addressing significant health disparities, OUTPACE offers an evidence-based, collaborative, and scalable approach to evaluating and optimizing QI initiatives. While traditional frameworks provide structure, OUTPACE offers nimbleness, responsiveness, and collaboration. Early phases (observe, uncover, and trial) help systems leverage lessons learned from prior programs and pilot new ideas efficiently. Personalize and accelerate enable tailored, small-scale testing to assess feasibility before broad investment. Finally, check and expand provide evidence-based steps to refine, validate, and scale programs to achieve QI goals.

## Summary of the Experience, Future Directions, and Challenges

OUTPACE was developed as an evolution of traditional QI methods, offering a generalizable framework for designing initiatives at local, regional, or national levels while allowing health care settings to tailor efforts to their specific ecosystems.

Although some signals detected by OUTPACE may not scale into meaningful improvements, the collaborative approach is intended to facilitate early detection of unanticipated consequences of pilot programs that could hinder success at scale.

The structured OUTPACE approach is designed to increase the likelihood of success of nascent QI initiatives by providing replicable, evidence-based steps for design and evaluation. Though developed for cardiovascular programs, we strongly encourage future QI initiatives focused on other clinical conditions to apply and report their experience with OUTPACE. Importantly, OUTPACE promotes expert collaboration, iterating and piloting with local teams and dedicated resources, and sharing of lessons learned before full-scale implementation.

## Article Information

### Acknowledgments

Statement on American Heart Association Journals’ Transparency and Openness Promotion Guidelines: data available upon request from the authors.

### Sources of Funding

The American Heart Association.

### Disclosures

M. Bolles, C. Rutan, M. Congdon, K. Overton, L. Serdynski, and K. Thomas and Drs Elkind and Jessup are employees of the American Heart Association. H.M. Alger is an employee of Anumana, Inc. Dr Joynt Maddox receives research support from the National Heart, Lung, and Blood Institute (grants R01HL143421 and R01HL164561), the National Institute of Nursing Research (grant U01NR020555), the National Institute on Aging (grant R01AG089215), the National Center for Advancing Translational Sciences (grant UL1TR002345), Schmidt Futures, Arnold Foundation, and the Missouri Foundation for Health. She serves as an Associate Editor for the *Journal of the American Medical Association*. She previously (through May 2023) served on the Health Policy Advisory Council for the Centene Corporation (St. Louis, MO) and received research funding from Humana (through December 2022). Dr Lewsey receives grant funding from the Winn Career Development Award. Dr Ndumele receives grant funding from the National Institutes of Health and the American Heart Association. Dr Fonarow reports consulting for Abbott, Amgen, AstraZeneca, Bayer, Boehringer Ingelheim, Cytokinetics, Eli Lilly, Johnson & Johnson, Medtronic, Merck, Novartis, and Pfizer. The other authors report no conflicts.

### Supplemental Material

Figures S1 and S2

## Supplementary Material
