# Supplementary material for: Introducing the OUTPACE Framework for Health Care Quality Improvement
Source: Circ Cardiovasc Qual Outcomes. 2025 Oct 21;18(12):e012211. doi: 10.1161/CIRCOUTCOMES.125.012211 (PMC12704659; doi:10.1161/CIRCOUTCOMES.125.012211)
Supplement: Supplementary file 1 [file hcq-18-e012211-s001.pdf]

Care Innovations: Introducing the OUTPACE Framework for Health Care Quality Improvement

Bolles, M. et al. 2025 Circulation Cardiovascular Care and Outcomes

Supplemental Data

Two case studies are presented to demonstrate the application of the OUTPACE framework to the development of a quality improvement (QI) initiative: Target: Aortic Stenosis (Target:AS, Supplemental Figure 1) and IMPLEMENT-HF (I-HF, Supplemental Figure 2). The OUTPACE framework was developed in observation of these case studies, as viewed through the lens of decades of experience and expertise among AHA volunteers and staff. These case studies were selected as they exemplify some of the key features that distinguish OUTPACE from existing frameworks.

While built on the steps of prior QI frameworks, OUTPACE specifically includes piloting and rapid cycle innovation and iteration before scaling to a broader QI initiative. This is demonstrated with the work that was done in the Target:AS case study (Supplemental Figure 1). This program did not intentionally follow the structured OUTPACE framework, instead the experience with Target:AS highlighted actions that informed the development of OUTPACE. Specifically, the Target:AS program paused after a stage that would be described as the “Personalize” stage, to return to an “Uncover” stage for additional evidence-gathering, prior to broadening the initiative.

Further, OUTPACE’s principle of collaborative learning, expert engagement, and iterating is highlighted in the IMPLEMENT-HF (I:HF) case study (Supplemental Figure 2). Similar to Target:AS, the experience with the I:HF initiative informed the development of OUTPACE, which is illustrated with the I-HF program returning to “Uncover”, “Personalize”, and “Accelerate”

during different stages of the initiative. The lessons learned from TAS and I-HF helped inform the development of OUTPACE.

Components of these steps are often used within the existing QI frameworks, whether they are codified within the approaches. Through detailed observation of the TAS and I-HF initiatives, the authors identified key QI activities that supported the programs' successes with the resources available (e.g., time and financing) and framed the steps within the OUTPACE framework to ensure that each step is expressly considered before, during, and at the completion of the QI initiative.

Supplemental Figure 1: Case Study: Evolution of the Target:Aortic Stenosis™ program informed by the OUTPACE Framework

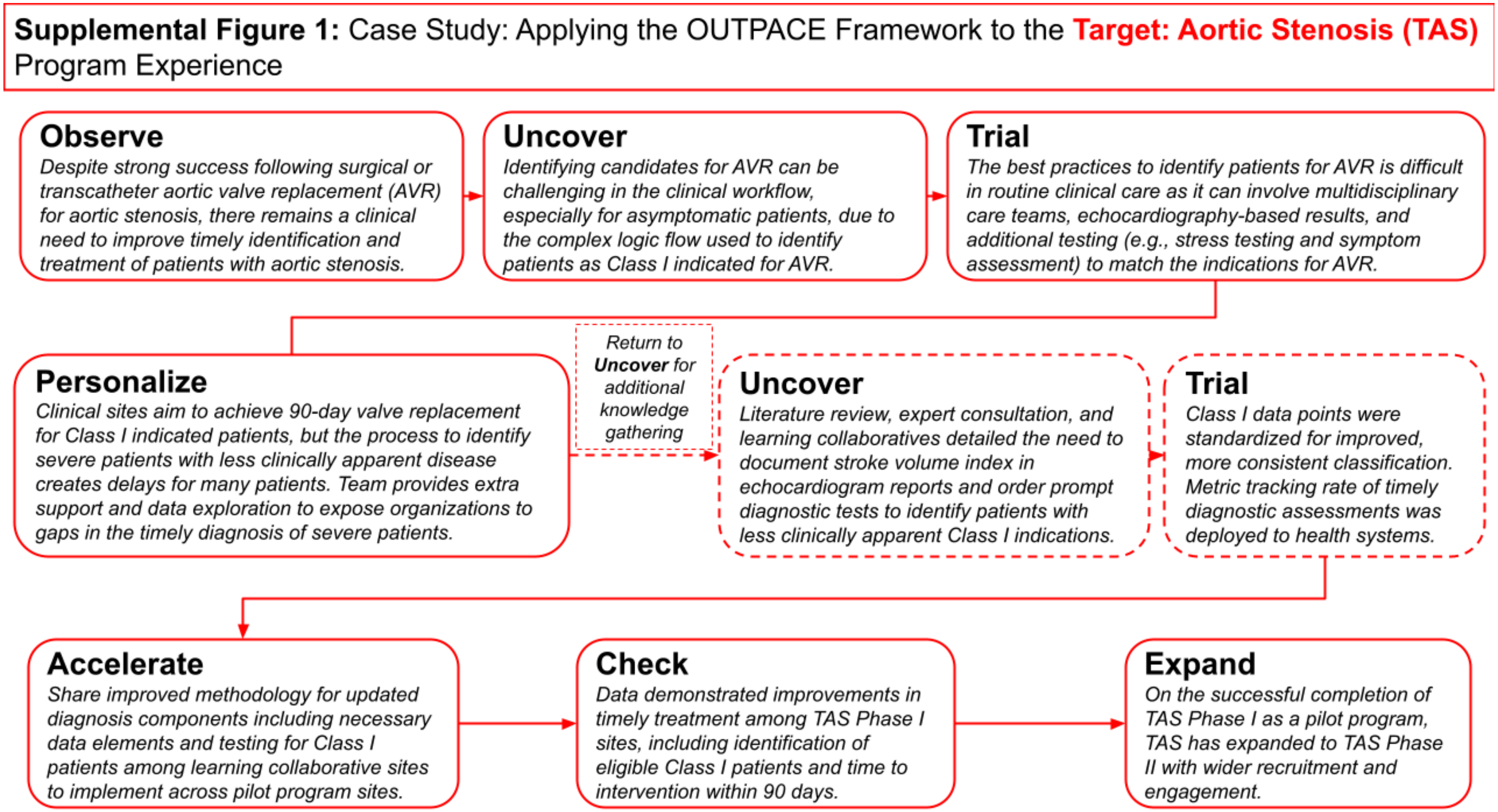

Note: Solid arrows indicate advancement to the next phase; dashed outlines and arrows indicate optional return to prior phase(s). Please visit https://www.heart.org/en/professional/quality-improvement/target-aortic-stenosis for more information and available resources about the Target: Aortic Stenosis program.

Abbreviations: AS: Aortic Stenosis; AVR: Aortic Valve Replacement

Supplemental Figure 2: Case Study: Evolution of the IMPLEMENT-HF™ program informed by the OUTPACE Framework

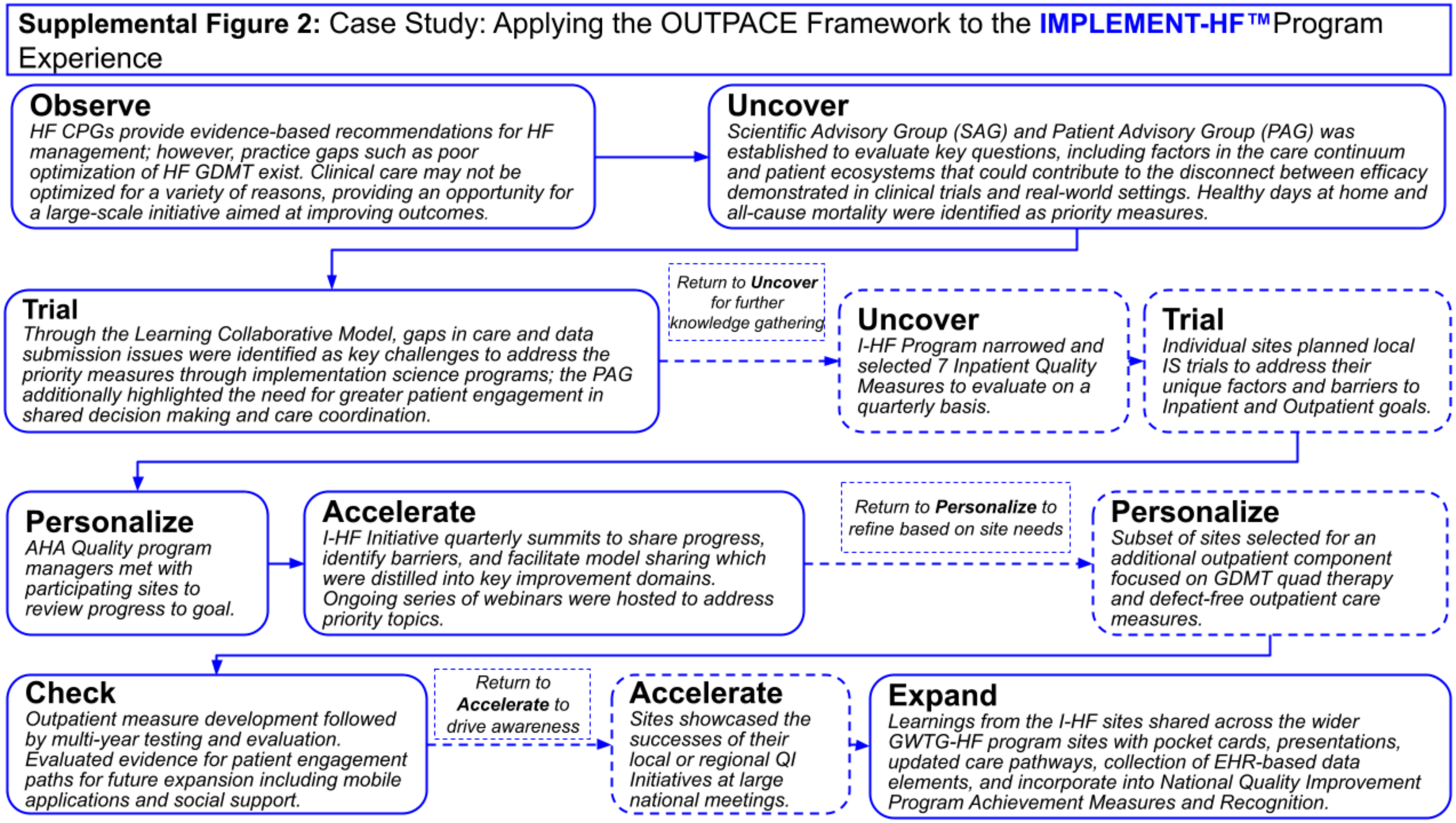

Note: Solid arrows indicate advancement to the next phase; dashed outlines and arrows indicate optional return to prior phase(s).
Please visit
https://www.heart.org/en/professional/quality-improvement/get-with-the-guidelines/get-with-the-guidelines-heart-failure/implement-hf
for more information and available resources about the IMPLEMENT-HF program.
Abbreviations: CPG: Clinical Practice Guidelines; GDMT: Guideline Directed Medical Therapy; HF: Heart Failure; IHF: IMPLEMENT-HF; IS: Implementation Science; PAG: Patient Advisory Group; SAG: Scientific Advisory Group
